# Introduction of ECT Discharge Summary- a Quality Improvement Project to Improve Communication Between Treating and Referring Clinicians and Aiming Better Patient Care

**DOI:** 10.1192/bjo.2022.264

**Published:** 2022-06-20

**Authors:** Ayesha Ahmad, Adnan Khan, Yetunde Abiodun

**Affiliations:** Essex Partnership University NHS Foundation Trust, Chelmsford, United Kingdom

## Abstract

**Aims:**

There was an initial QIP done in 2019 highlighting the deviation and deficits from ECTAS Guidelines. We tried to make positive change by introducing checklists and assessments. We were able to reach our goal but noted that collating information was a time taking task. More ever, documents were not always accessible as patients come to Mid Essex ECT clinic from other parts of Trust as well as from Private inpatient settings which meant that we did not have records for those patients. We noticed clear lack of communication between out of area referring and treating clinician regarding treating team's view about patient progress, assessment results and recommendations for future ECT need, which we thought could be improved by generating discharge summary of each patient as end of treatment.

**Methods:**

We conducted retrospective audit for all treated patients in ECT clinic in 2020(n = 18,re-audited in 2021 and 2022(n = 13) to see documentation, including Consent documents; Form 5(consent) in case of capacity, T4/T6/S62, Documentation of Memory assessment, as well as MADRS assessment before and during the procedure. We started generating ECT discharge summaries in November 2021 and collected data for all patients (13) till 6th February 2022.

**Results:**

Documentation of Legal Status: 5% in 2020 vs.100% in 2021* and 2022

Written consent /form 5: 95% in 2020 vs. 100% in 2021* and 2022

Documented Mini-ACE: 5% in 2020 vs. 100% in 2021* and 2022

Doc. MADRS assessment; 0% in 2020 vs. 100% in 2021* and 2022

*(excluding patients who did not complete the treatment)

**Conclusion:**

The audit results of 2020 showed improvement however assessments done during treatment were not accessible to referring clinicians or to patients. Introduction of discharge summary helped to give snapshot of patient's weekly progress, weekly objective assessment scores which helped the referring clinicians to get idea about patient's improvement and resulted in improved communication as well as patient and carer satisfaction.

Small actions can have big impact on the way patient care is delivered. We believe that going through process of auditing helped us to improve our practice and make a positive change in terms of delivering better care.

